# Improved magnetostriction in Galfenol alloys by aligning crystal growth direction along easy magnetization axis

**DOI:** 10.1038/s41598-020-77058-2

**Published:** 2020-11-18

**Authors:** Chao Zhou, Yapeng Liu, Kaiyun Chen, Zhiyong Dai, Tianyu Ma, Yu Wang, Shuai Ren, Junkai Deng, Rui Zhang, Fanghua Tian, Yin Zhang, Hao Zeng, Sen Yang

**Affiliations:** 1 School of Physics, MOE Key Laboratory for Nonequilibrium Synthesis and Modulation of Condensed Matter, State Key Laboratory for Mechanical Behavior of Materials, Xi’an Jiaotong University, Xi’an, 710049 China; 2 Frontier Institute of Science and Technology, Xi’an Jiaotong University, Xi’an, 710049 China; 3 Department of Physics, University at Buffalo, State University of New York, Buffalo, NY 14260 USA

**Keywords:** Materials science, Physics

## Abstract

Galfenol (Iron-gallium) alloys have attracted significant attention as the promising magnetostrictive materials. However, the as-cast Galfenols exhibit the magnetostriction within the range of 20–60 ppm, far below the requirements of high-resolution functional devices. Here, based on the geometric crystallographic relationship, we propose to utilize the 90°-domain switching to improve the magnetostriction of Galfenols by tuning the crystal growth direction (CGD) along the easy magnetization axis (EMA). Our first-principles calculations demonstrate that Pt doping can tune the CGD of Galfenol from [110] to [100], conforming to the EMA. Then, it is experimentally verified in the (Fe_0.83_Ga_0*.*17_)_100*−x*_Pt_*x*_ (*x* = 0, 0.2, 0.4, 0.6, 0.8 and 1.0) alloys and the magnetostriction is greatly improved from 39 ppm (*x* = 0, as-cast) to 103 ppm (*x* = 0.8, as-cast) and 188 ppm (*x* = 0.8, directionally solidified), accompanying with the increasing CGD alignment along [100]. The present study provides a novel approach to design and develop high-performance magnetostrictive materials.

## Introduction

The magnetostrictive effect, which realizes the conversion between mechanical energy and magnetic energy, provides the fundamental physics for important functional devices, such as sensors (e.g., stress/force/position sensors), actuators (e.g., linear motors) and transducers (e.g., energy harvesting devices)^1,2^.

Among the promising magnetostrictive materials, Fe–Ga (Galfenol) alloys have garnered significant research attention due to the excellent magnetoelastic properties and desirable comprehensive performance for mechanical working, such as excellent ductility and high mechanical strength^3–7^. Much effort has been dedicated to improving the magnetostriction of FeGa alloys, including enhancing the local magneto-crystalline anisotropy from the introduction of the tetragonal nanoheterogeneities (m-D03 phase)^8–14^, application of prestress and magnetic annealing^3,5,6,15–17^, etc. But these methods result in design complexities and high fabrication cost.

**Figure 1:**
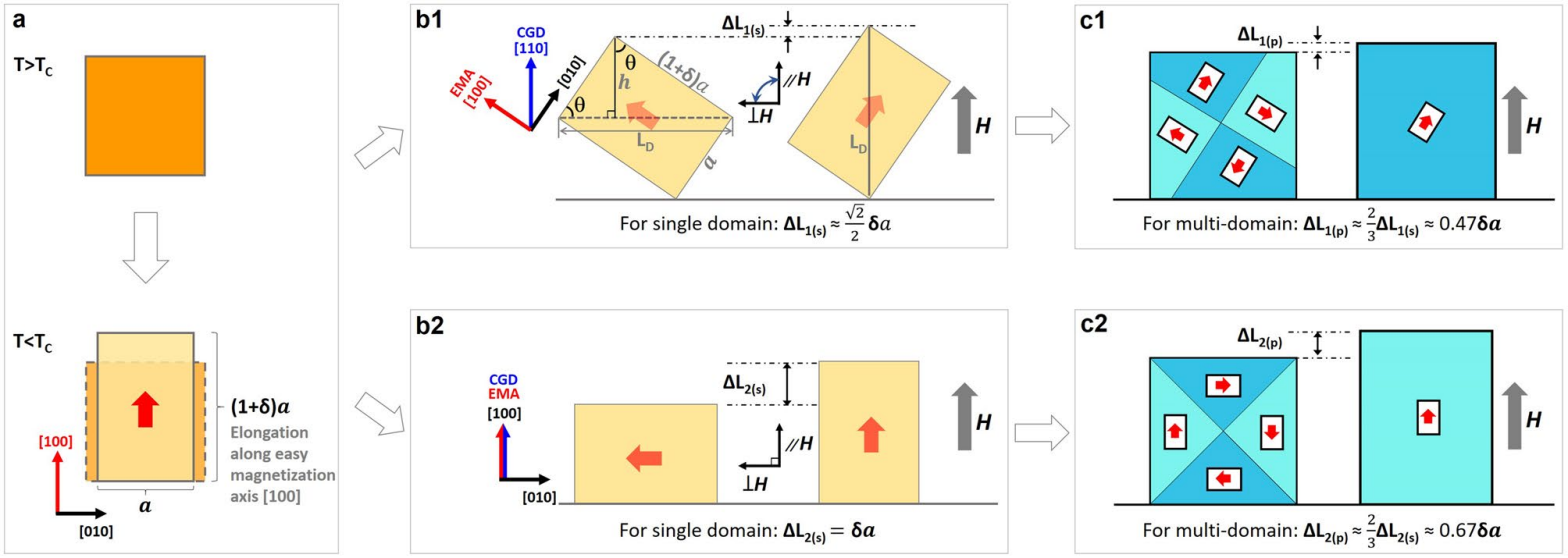
The schematic illustrations of the magnetostriction from two cases of 90°-domain switching of FeGa: the crystal growth direction (CGD) is same with/different from the easy magnetization axis (EMA). (**a**) The normal elongation of the crystal along EMA below Curie temperature; (**b1**) the single domain switching with the CGD deviating from the EMA (CGD along [110] and EMA along [100]); (**b2**) the single domain switching with the CGD parallel to the EMA (CGD along [100] and EMA along [100]); (**c1**) the multi-domain case for (b1); (**c2**) the multi-domain case for (**b2**). The red arrows represent the direction of EMA.

The magnetostrictive effect originates from the lattice distortion caused by magnetoelastic coupling; such lattice distortion gives rise to the distorted magnetic domain along the easy magnetization axis (EMA). The magnitude of measured magnetostriction (only Joule magnetostriction is considered here^18^) is mainly determined from the 90°-domain switching^19^, and only the change of domain distortion component along the external field, which is usually parallel to the crystal growth direction (CGD), contributes to the measured magnetostriction during the switching of 90°-domain, as schematically illustrated in Fig. 1. Therefore, the CGD conforming to EMA (parallel to domain distortion) and the CGD deviating from the EMA, deliver different magnetostrictions.

In this work, we propose to utilize the 90°-domain switching to improve the magnetostriction of Galfenols by tuning the CGD along the easy magnetization axis (EMA). We shall show in order the 90°-domain-switching based magnetostrictive mechanism in ferromagnetic materials, the density functional theory (DFT) calculations to predict the CGD tuning by elemental doping, and the improved magnetostriction in FeGa alloys. Based on the results of the DFT calculations, we have synthesized Pt-doped FeGa alloys—(Fe_0.83_Ga_0*.*17_)_100*−x*_Pt_*x*_ (*x* = 0, 0.2, 0.4, 0.6, 0.8 and 1.0) and demonstrated the enhancement effect in magnetostriction with the CGD aligning along EMA. The proposed methodology provides a general pathway to design high-performance magnetostrictive materials.

## Results

### The proposed magnetostrictive model based on 90°-domain switching

The Fe_1−*x*_Ga_*x*_ (*x* < 0.2) possesses a body-centered cubic (BCC) A2 structure, with Fe and Ga atoms distributed disorderedly^5^; the EMA is along <100>^20^ and the CGD is along <110>^21^. Herein, we have considered the EMA along [100] and CGD along [110] to conveniently illustrate and describe the magnetostrictive model, which does not influence the proposed methodology. In the case of CGD deviating from EMA, the corresponding magnetostrictive process is illustrated in Fig. 1a,b1,c1. Figure 1a presents the normal elongation of the crystal along EMA below Curie temperature, i.e., *δa*, where *a* refers to the lattice constant and *δ* represents the elongation coefficient of the lattice distortion. The 90°-domain switching ends with the external magnetic field (***H***) parallel to the CGD [110]. If a single domain state is considered (Fig. 1b1), the magnetostriction along ***H*** is calculated to be ~ $\frac{\sqrt 2 }{2}$. *δa*. The calculations of the magnetostriction (∆L_1(s)_ in Fig. 1b1) are as below:

1 $$ \begin{aligned} \Delta L_{1(S)} & = L_{D} - 2 \cdot h \\ & = [a \cdot \cos \theta + (1 + \delta ) \cdot a \cdot \sin \theta ] - 2 \cdot a \cdot \sin \theta \\ & = a \cdot [\cos \theta + (1 + \delta ) \cdot \sin \theta - 2 \cdot \sin \theta ] \\ & = a \cdot [\delta \cdot \sin \theta + (\cos \theta - \sin \theta )] \\ \end{aligned} $$

The experimentally measured magnetostriction (10–100 ppm) demonstrates that *δ* is of the order of magnitude of 10^−4^. Therefore, the angle θ is approximately equal to 45°. Consequently, the magnetostriction ∆L_1(s)_ is:

2 $$ \begin{aligned} \Delta L_{1(S)} & \approx a \cdot [\delta \cdot \sin 45^{ \circ } + (\cos 45^{ \circ } - \sin 45^{ \circ } )] \\ & = a \cdot \delta \cdot \sin 45^{ \circ } \\ & = \frac{\sqrt 2 }{2}\delta a \\ \end{aligned} $$

If a multi-domain state is considered (Fig. 1c1), the magnetostriction is equal to the elongation multiplied with a domain configuration factor. For simplicity, the domain configuration factor is fixed as 2/3, corresponding to the homogeneous distribution of domains along six axes of the BCC structure. Hence, the magnetostriction for the multi-domain state is calculated to be ~ 0.47*δ*a.

In another case, if the CGD is parallel to the EMA along [100], the shape distortion of the 90°-domain switching completely contributes to the measured magnetostriction. This is illustrated in Fig. 1a,b2,c2. The 90°-domain switching ends with the external magnetic field ***H*** parallel to the EMA [100]. For the 90° switching of a single domain, the value of magnetostriction is equal to *δa* (Fig. 1b2). If a multi-domain state is considered (Fig. 1c2), the calculated magnetostriction is ~ 0.67*δa*, which is obtained by multiplying the multi-domain factor (2/3). In brief, if the CGD [110] can be tuned to the EMA [100], a large improvement in magnetostriction is expected.

However, the experimental realization of such domain switching process remains a challenge. Given the relationship between surface formation energy and the growth surface (growth plane) that correlates to the crystal growth direction, the preferred growth direction can be determined from the plane with the lowest surface formation energy^22,23^. Therefore, if elemental doping can tune the plane with the lowest surface formation energy of FeGa alloy, from (110) to (100), it is expected to improve the magnetostriction.

### The DFT calculations

**Figure 2:**
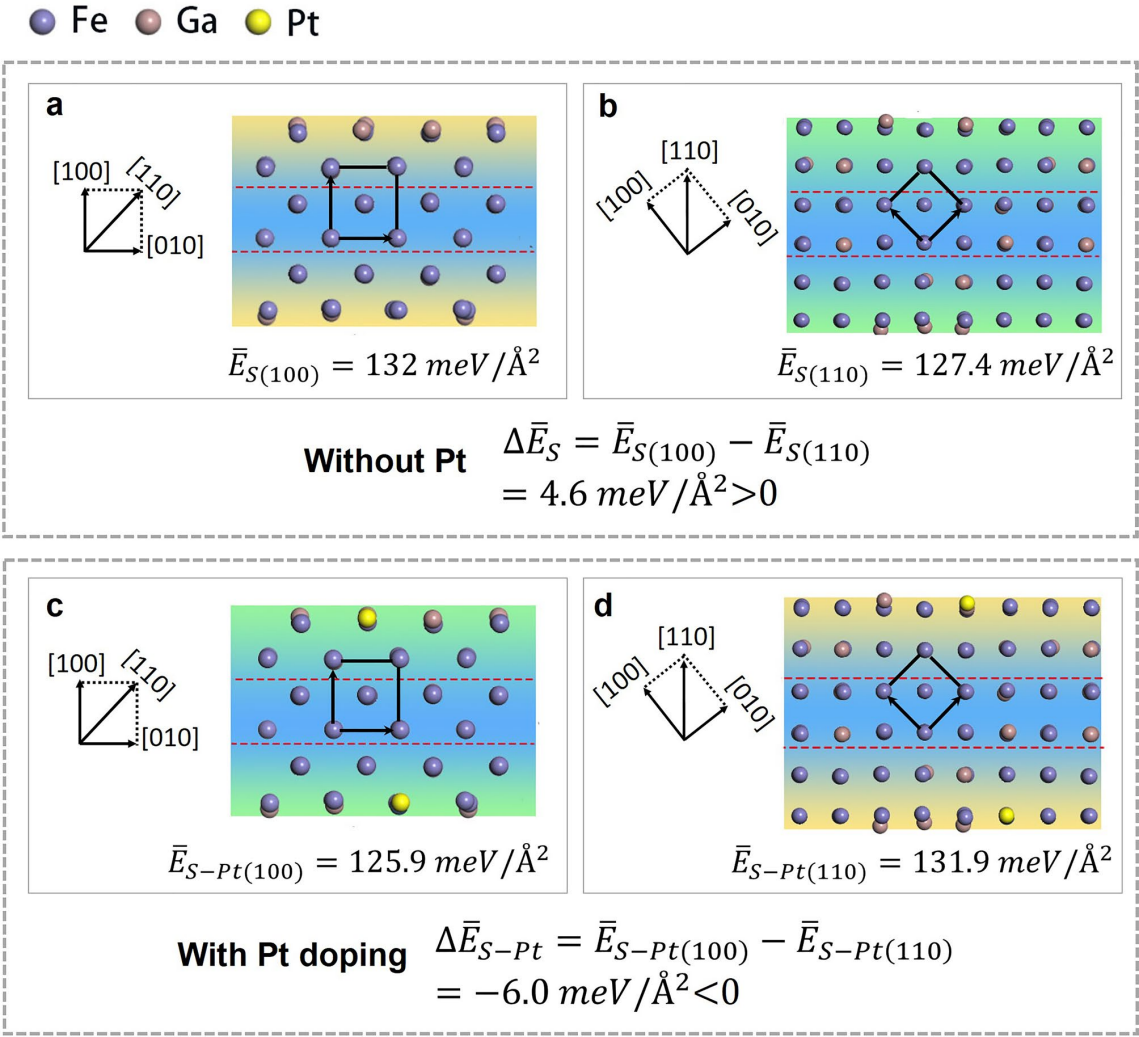
The surface formation energies determined by DFT calculations: side view of atomic structures for (**a**) FeGa alloy slab along [100], (**b**) FeGa alloy slab along [110], (**c**) FeGa alloy slab along [100] with Pt doping on its surface, (**d**) FeGa alloy slab along [110] with Pt doping on its surface. Orange regions correspond to the higher energy surfaces, whereas the green regions represent the lower energy surfaces. The blue regions refer to the interior of crystals.

In the following, the DFT calculations are carried out to demonstrate that Pt doping into FeGa crystal tunes the plane with the lowest surface formation energy from (110) to (100). Considering the large spin–orbit coupling of the 5*d* element Pt and the potentially strong interactions between Pt and Fe, which possesses strong spin polarization, Pt is chosen as the doping element to tune the CGD of FeGa crystal^24^. Since Fe_0.83_Ga_0.17_ have exhibited relatively large magnetostriction within a wide temperature range and can endure large tensile stresses, our samples are fixed as (Fe_0.83_Ga_0.17_)_100−*x*_Pt_*x*_, where Pt substitutes the Fe and Ga sites within the A2 matrix^3^. Owing to the random disorder A2 structures of Fe_0.83_Ga_0.17_^25^, special quasirandom structure (SQS) approximation was used to construct a 3 × 3 × 3 BCC supercell, which has the stoichiometry of Fe_45_Ga_9_, whereas the nominal composition is Fe_0.83_Ga_0.17_^26^. The bulk structure of Fe_45_Ga_9_ was completely relaxed. Based on the relaxed bulk structure, six-layered atomic slabs of FeGa, perpendicular to (100) and (110) surfaces, were built with different surface configurations. Only one Pt atom was doped into each surface of FeGa slabs to examine the influence of Pt. The total energy and surface area of the above-mentioned structures are summarized in Table 1. The surface formation energy can be defined as ${E}_{s}=\frac{{E}_{slab}-{E}_{bulk}}{2S}$, where *E*_*slab*_ and *E*_*bulk*_ refer to the total energy of slab and bulk, respectively, and *S* represents the surface area. Considering multiple surface configurations, *E*_*S*_ is determined by an average value of all slabs. As illustrated in Fig. 2, only one slab is depicted as a representative. The surface energy of (100) and (110) in FeGa slab is $\overline{E}_{{S\left( {100} \right)}} = 132$ meV/Å^2^ and $\overline{E}_{{S\left( {110} \right)}} = 127.4$ meV/Å^2^, respectively (Fig. 2a,b). The difference between the surface energy of (100) and (110) is ${\Delta }\overline{E}_{S} = \overline{E}_{{S\left( {100} \right)}} - \overline{E}_{{S\left( {110} \right)}} = 4.6$ meV/Å^2^, which implies that (110) is more stable than (100) and easily forms during solidification. The replacement of Fe or Ga by Pt on the surface of the above slabs hardly changes the atomic structure, as shown in Fig. 2c,d. However, the surface formation energy of (100) and (110) is changed to $\overline{E}_{{S - Pt\left( {100} \right)}} = 125.9$ meV/Å^2^ and $\overline{E}_{{S - Pt\left( {110} \right)}} = 131.9$ meV/Å^2^, respectively. Hence, the difference in surface formation energies becomes ${\Delta }\overline{E}_{S - Pt} = \overline{E}_{{S - Pt\left( {100} \right)}} - \overline{E}_{{S - Pt\left( {110} \right)}} = - 6.0$ meV/Å^2^. Apparently, the surface formation energy relationship is reversed, which indicates that (100) is more stable than (110) and becomes a preferred growth plane after Pt doping. The DFT calculations indicate that doping of Pt can tune the CGD of FeGa from [110] to [100] (parallel to the EMA), which is expected to improve the magnetostriction, as depicted in Fig. 1.

**Table 1:** Total energy of different slab structures before and after Pt doping.

	Surface	No	1	2	3	4	Average *E*	${\stackrel{-}{E}}_{s}$(eV/Å^2^)
Fe–Ga	(1 0 0)	E (eV)	− 379.5764	− 382.6708	− 384.1361	− 380.7047	− 381.7720	0.1320
		S (Å^2^)	68.1251	69.2346	69.2287	68.1038	68.6730	
	(1 1 0)	E (eV)	− 773.8652	− 773.6429	− 773.8266		− 773.7782	0.1274
		S (Å^2^)	102.2652	102.3563	101.9178		102.1798	
Fe–Ga–Pt	(1 0 0)	E (eV)	− 382.9563	− 384.4188	− 386.0369	− 382.5636	− 383.9939	0.1259
		S (Å^2^)	8.2348	8.3234	8.3195	8.2650	8.2857	
	(1 1 0)	E (eV)	− 775.7081	− 775.3140	− 775.8527		− 775.6249	0.1319
		S (Å^2^)	102.4392	102.7928	102.3055		102.5125

### X-ray diffraction analysis

**Figure 3:**
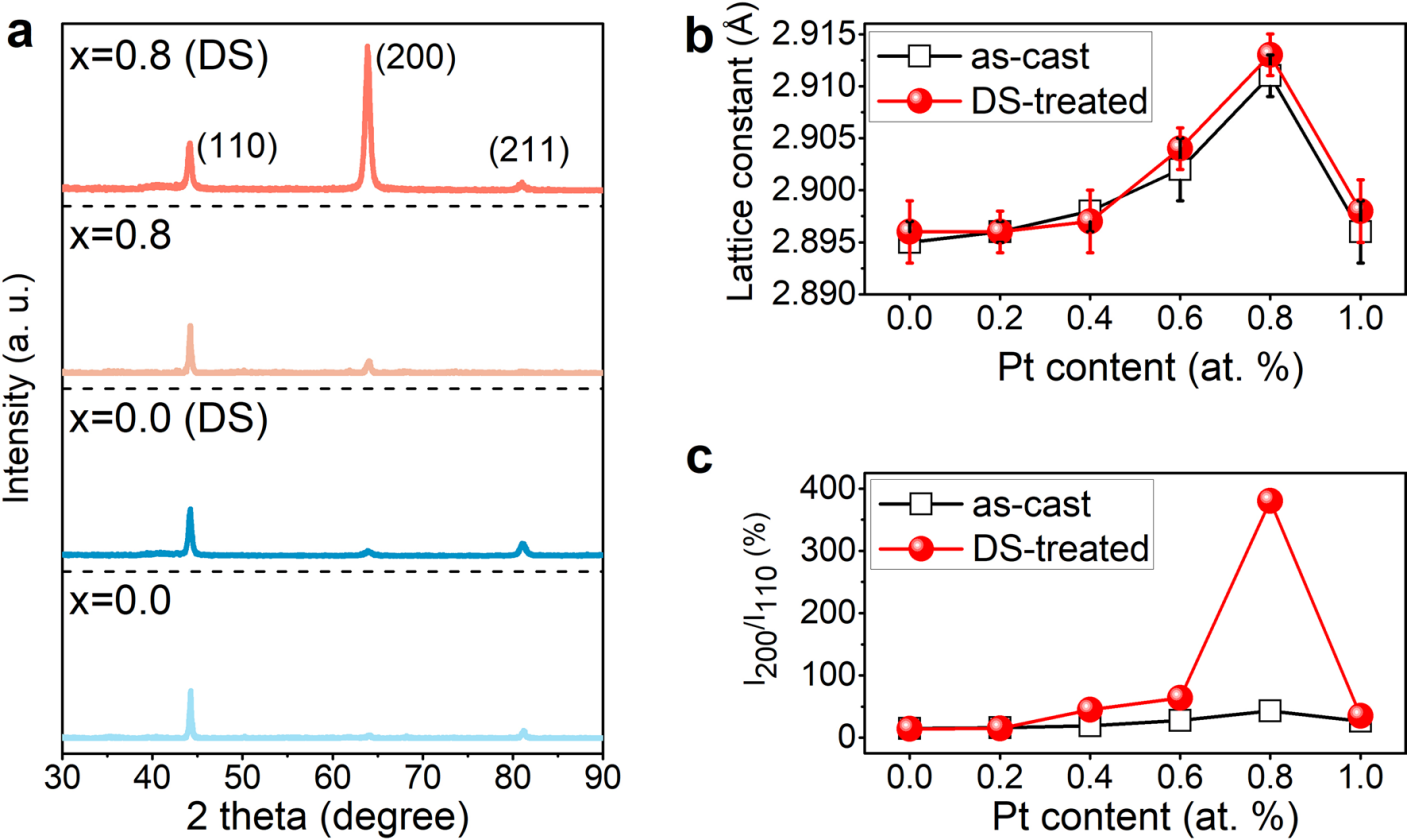
XRD patterns of (Fe_0.83_Ga_0.17_)_100*−x*_Pt_*x*_ alloys: (**a**) *x* = 0 and 0.8 for as-cast and DS-treated samples; (**b**) the Pt dependence of the intensity ratio between (200) peak and (110) peak for as-cast and DS-treated samples; (**c**) the Pt dependence of the lattice constant for as-cast and DS-treated samples.

For verification, we prepared both as-cast and directionally solidified (DS) Pt-doped FeGa crystals. The X-ray diffraction (XRD) patterns of the selected compositions are shown in Fig. 3a. The as-cast and the DS-treated samples exhibit only characteristic (110), (200) and (211) peaks within the 2theta range from 30° to 90°, demonstrating that pure body-centered cubic (BCC) A2 structure is retained after Pt doping^15,27^. Figure 3b shows the Pt content dependence of lattice constant. With the increase of Pt content, the lattice constants of both as-cast and DS-treated FeGa crystals increase to the maximum at *x* = 0.8 and then decrease at *x* = 1.0. Figure 3c presents the Pt content dependence of the intensity ratio (I_200_/I_110_) between (200) and (110) peaks. The maximum of I_200_/I_110_ appears at *x* = 0.8 for both as-cast and DS-treated crystals, 43.1% and 380.3%, respectively. When Pt content exceeds 0.8, I_200_/I_110_ decreases to 26.5% and 34.9% at *x* = 1.0, for as-cast and DS-treated samples, respectively. The decrease of the lattice constant and the orientation preference along [100] when Pt content exceeds 0.8, may be caused by the solubility limit of Pt in FeGa crystal. It is clear that before and after Pt doping, the samples maintain polycrystalline state, and the results of the intensity ratio of I_200_/I_110_ demonstrate the favorable growth direction along [100] with Pt doping up to 0.8 at.%, which is in good agreement with DFT calculations.

### Magnetostriction and magneto-crystalline anisotropic constant K_1_

**Figure 4:**
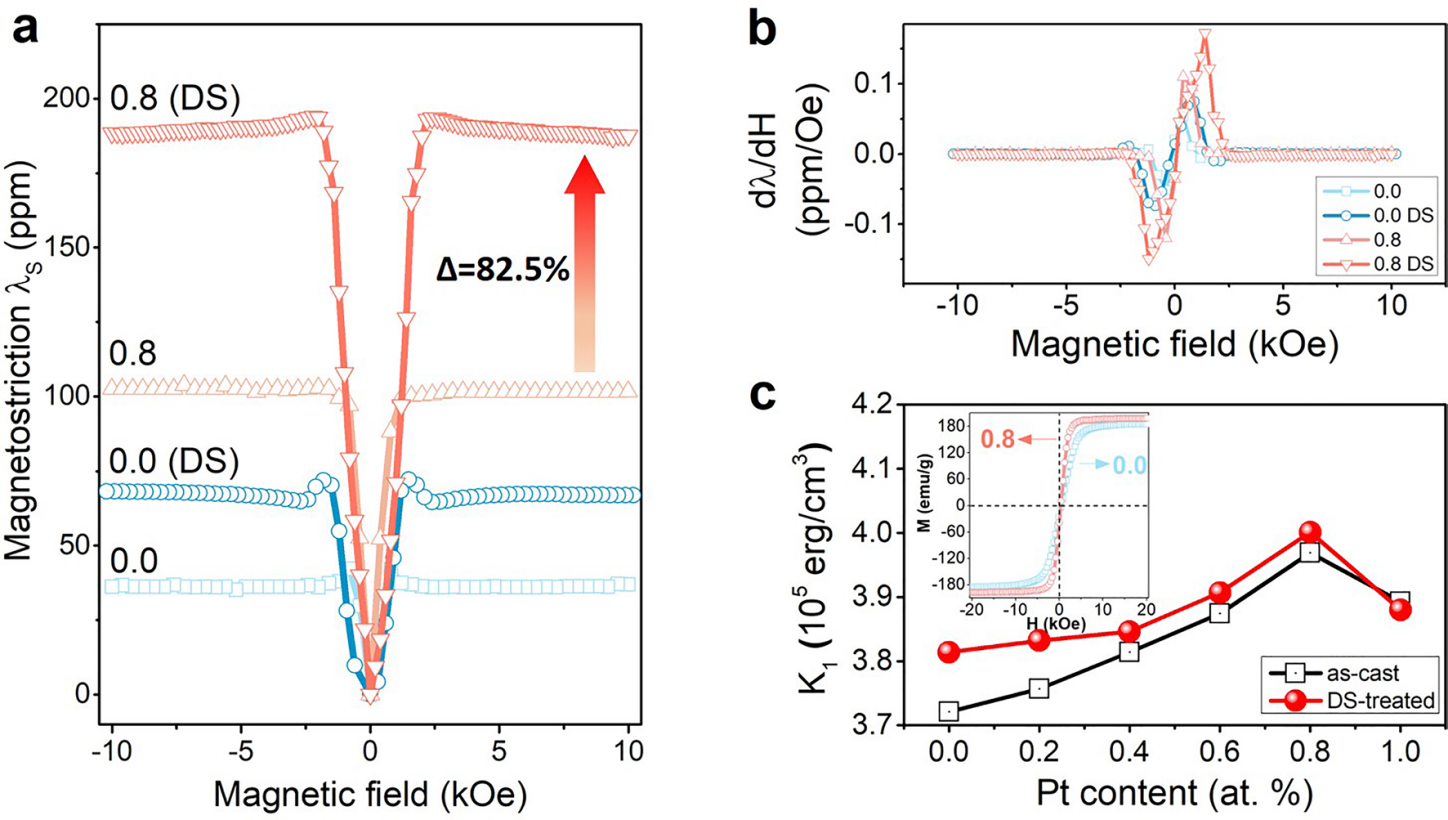
The magnetostriction curves and magneto-crystalline anisotropy constant K_1_ of (Fe_0.83_Ga_0.17_)_100*−x*_Pt_*x*_ alloys: (**a**) *x* = 0 and 0.8 for as-cast and DS-treated samples; (**b**) first order derivatives of magnetostriction over magnetic field (*x* = 0 and 0.8 for as-cast and DS-treated samples); (**c**) the calculated magneto-crystalline anisotropic constant K_1_ with the inset showing the ***M***–***H*** hysteresis loops (*x* = 0 and 0.8).

Figure 4a presents the Pt content dependent magnetostriction curves at room temperature. The un-doped FeGa sample (*x* = 0) shows the magnetostriction of 39 ppm, which is consistent with the previously reported values of 20–60 ppm and theoretical prediction^28^. The composition *x* = 0.8 shows the highest magnetostriction of 103 ppm for as-cast sample and 188 ppm for DS-treated sample. It should be noted that as for the trace-amount element doped FeGa polycrystalline alloys without treatment of magnetic annealing and prestress during measurement, the reported maximum value of magnetostriction (even DS-treated FeGa alloys) is 160 ppm^29–34^. Meanwhile, the Pt doping causes a higher magnetostriction without increasing the saturation field, which can be quantified as d**λ**/d***H***, as shown in Fig. 4b. For as-cast and DS-treated samples, the largest d**λ**/d***H*** appears at *x* = 0.8 and reaches the maximum value of 0.11 ppm/Oe and 0.17 ppm/Oe, respectively. This feature is also desirable in practical applications. The higher the value of d**λ**/d***H*** is, the lower field is needed to trigger large magnetostriction. In practical applications, the magnetic materials with higher value of d**λ**/d***H*** can help realize the miniaturization of devices.

Because the samples are polycrystalline, the magneto-crystalline anisotropic constant K_1_ cannot be measured directly. Using the methods proposed by Vazquez et al. and Andreev et al.^35,36^, the magneto-crystalline anisotropic constant K_1_ is calculated from the magnetization as a function of magnetic field (Fig. 4c, details can be referred to the supplementary materials). For our samples, the calculated K_1_ ranges 3.7–4.0 × 10^5^ erg/cm^3^, while Rafique. S et al. have reported that the K_1_ of FeGa alloy ranges 3–7 × 10^4^ J/m^3^^37^. Considering the relation that 1 erg/cm^3^ = 10^–1^ J/m^3^, the values of calculated K_1_ for our FeGa samples are in good agreement with the previous reports. From the inset of Fig. 4c, it can be seen that compared with *x* = 0.8, the sample of *x* = 0 is harder to be magnetized, reflected from both the saturated magnetization and the slope of the curve. This result agrees well with Fig. 1c1,c2. The FeGa–*x*Pt alloy of *x* = 0.8 exhibits the highest magneto-crystalline anisotropic constant K_1_ of 3.97 ∗ 10^5^ erg/cm^3^ and 4.00 ∗ 10^5^ erg/cm^3^ for as-cast and DS-treated samples, respectively. The trend of K_1_ variation with Pt content is also consistent with the intensity ratio I_200_/I_110_. The increase of K_1_ with the increase of Pt content, up to *x* = 0.8, demonstrates the increase of magneto-crystalline anisotropy and, consequently, the increase of *δ* (Fig. 1), which further improves the magnetostriction.

### Discussion

It should be noticed that, for the same composition, e.g., *x* = 0, the DS-treated sample and the as-cast sample exhibit different magnetostrictions while there are no obvious differences in I_200_/I_110_ (Figs. 3c, 4a). Considering that DS is one type of heat treatment process, the differences between *x* = 0 (as-cast) and *x* = 0 (DS-treated) are probably due to the nanoheterogeneities (m-D03 phase nano-inclusions or precipitates) that have been studied extensively^11–14,38^.

Last but not least, let us return to the previously reported methods to improve the magnetostriction of FeGa alloys. The XRD patterns of the FeGa alloys prepared by magnetic annealing^15^, melt-spinning^9,39^, and single crystal growth^27^ all indicate the positive correlation between the magnetostriction and the preferred growth direction along [100]. This validates our proposed model and indicates the presence of a common physical mechanism behind these different approaches. Compared with previous work, the fundamental scientific objective of the present work is to tune the intrinsic factor to enhance the magnetostriction, without considering complex treatments and measurement auxiliary conditions. Besides, with the assistance of DFT calculations, the design and fabrication of high-performance FeGa alloys will be more efficient.

In conclusion, based on a crystallographic geometric model, we propose an approach to improving the magnetostriction of FeGa alloys by aligning the crystal growth direction with the easy magnetization axis, and the DFT calculations indicate that the doping of Pt can facilitate such process through tuning the crystal surface formation energy. Furthermore, the proposed model is experimentally verified: accompanying with the increasing preference of the crystal growth along [100] direction, the magnetostriction of FeGa with Pt doping is greatly improved from 39 ppm (*x* = 0) to 103 ppm (*x* = 0.8) and 188 ppm (*x* = 0.8, DS). The present study provides an effective approach to explore and design high-performance magnetostrictive materials.

## Methods

### Sample preparation and characterization

The (Fe_0.83_Ga_0*.*17_)_100*−x*_Pt_*x*_ polycrystalline samples (x represents the atomic percentages; *x* = 0, 0.2, 0.4, 0.6, 0.8 and 1.0) were prepared by using high purity metals of Fe (99.95%), Ga (99.99%) and Pt (99.99%) by arc-melting techniques under argon atmosphere. Considering the lower melting point of Ga, excessive 1 mol% of Ga was added to compensate the losses during the melting process. To ensure compositional homogeneity, each sample (~ 7 g) was melted four times and the weight loss of the ingots was less than 1%. The as-cast ingots were sectioned into slices with a thickness of 1 mm by wire-cutting and sealed into a quartz tube, filled with argon gas, followed by a heat treatment at 1000 °C for 3 h, and then quenched into water. The directionally solidified samples were prepared at 1680 °C with the pulling rate of 5 µm/s. The X-ray diffraction (XRD) patterns were measured by using a Bruker D8 ADVANCE Diffractometer (*Cu–Kα*, *λ* = 1.5406 Å) and the lattice constants of FeGa–Pt alloys were calculated by using Nielsen extrapolation method.

### Property measurements

The magnetic characterization was carried out on the superconducting quantum interference device-vibrating sample magnetometer (MPMS-SQUID VSM-094). The magnetostriction was tested with a standard strain gauge at room temperature.

### Computational methods

The Vienna Ab-initio Simulation Package (VASP) was used to conduct DFT calculations with the projector augmented wave method^40^. The spin-polarized generalized gradient approximation (GGA) with the Perdew–Burke–Ernzerhof (PBE) functional was adopted^41^. The kinetic energy cutoff for wavefunction expansion was set to 400 eV. Monkhorst–Pack k-point grid of 3 × 3 × 3 and 3 × 3 × 1 was used for bulk and slab supercell, respectively. Atomic positions and lattice constants were completely relaxed in bulk. For the slab models, two surface atomic layers were completely relaxed, but two middle atomic layers were fixed in lattice constants of the relaxed bulk model to simulate the surface influence. Both relaxations were terminated once the difference of energy and force was less than 10^–5^ eV and 0.01 eV/Å, respectively. The periodic boundary conditions were applied in calculations, while the vacuum space was added along the z-axis for slab model at least 20 Å to safely avoid artificial interaction between the periodic images.

## Supplementary information

Supplementary information.

## Data Availability

The data that support the findings of this study are available from the corresponding authors on request.
