# Supplementary material for: Improved magnetostriction in Galfenol alloys by aligning crystal growth direction along easy magnetization axis
Source: Sci Rep. 2020 Nov 18;10:20055. doi: 10.1038/s41598-020-77058-2 (PMC7674428; doi:10.1038/s41598-020-77058-2)
Supplement: Supplementary file 1 — Supplementary information. [file 41598_2020_77058_MOESM1_ESM.docx]

**Supplementary Information**

1. **Calculations of the magnetostriction in Fig. 1(b1) and the magnetostriction improvement by aligning the crystal growth direction (CGD) along the easy magnetization axis (EMA)**


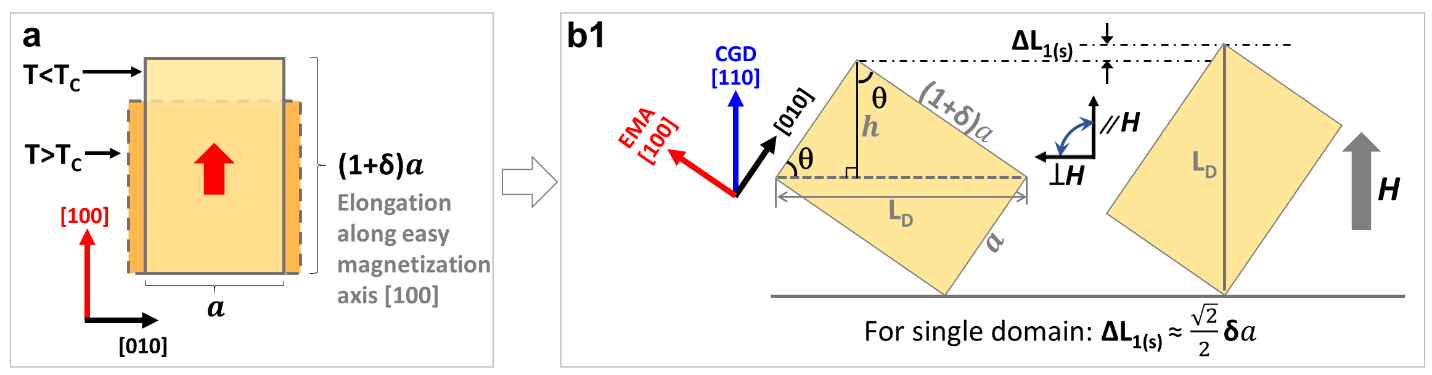


**Figure S1**. The schematic illustration of magnetostriction calculations of 90°-domain switching, ending with the external magnetic field ***H*** being parallel to the CGD [110] (EMA along [100])

The calculations of the magnetostriction (**ΔL_1(s)_**) in Fig. 1(b1) are as below:

$\begin{matrix} \Delta L_{1(S)}=L_{D}-2\cdot h \\ =[a\cdot\cos\theta+(1+\delta)\cdot a\cdot\sin\theta]-2\cdot a\cdot\sin\theta\\ =a\cdot[\cos\theta+(1+\delta)\cdot\sin\theta-2\cdot\sin\theta] \\ =a\cdot[\delta\cdot\sin\theta+(\cos\theta-\sin\theta)] \end{matrix}$ (1)

where δ denotes the elongation coefficient of the lattice distortion along the EMA. The experimentally measured magnetostriction (10~100 ppm) demonstrates that δ is of the order of magnitude of 10^-4^. Therefore, the angle θ is approximately equal to 45°. Consequently, the magnetostriction **ΔL_1(s)_** is:

$\begin{matrix} \Delta L_{1(S)}\approx a\cdot[\delta\cdot\sin45^{\circ}+(\cos45^{\circ}-\sin45^{\circ})] \\ =a\cdot\delta\cdot\sin45^{\circ} \\ =\frac{\sqrt{2}}{2}\delta a \end{matrix}$ (2)

The 90°-domain switching for the multi-domain state with the CGD along [110] delivers the magnetostriction of ~0.47δ*a* [Fig. 1(c1)] and the 90°-domain switching for the single domain state, with the CGD along [100], delivers the magnetostriction of δ*a* [Fig. 1(b2)]. Theoretically, if a multi-domain state with the CGD along [110] can be tailored to single domain state with the CGD along [100], the increase rate (**ΔL_I_**) of the magnetostriction can reach 112.8%, as shown below:

$\Delta L_{I}=\frac{\delta\cdot a-0.47\cdot\delta\cdot a}{0.47\cdot\delta\cdot a}=\frac{0.53}{0.47}=112.8\%$ (3)

1. **XRD results of as-cast and DS-treated samples**

**
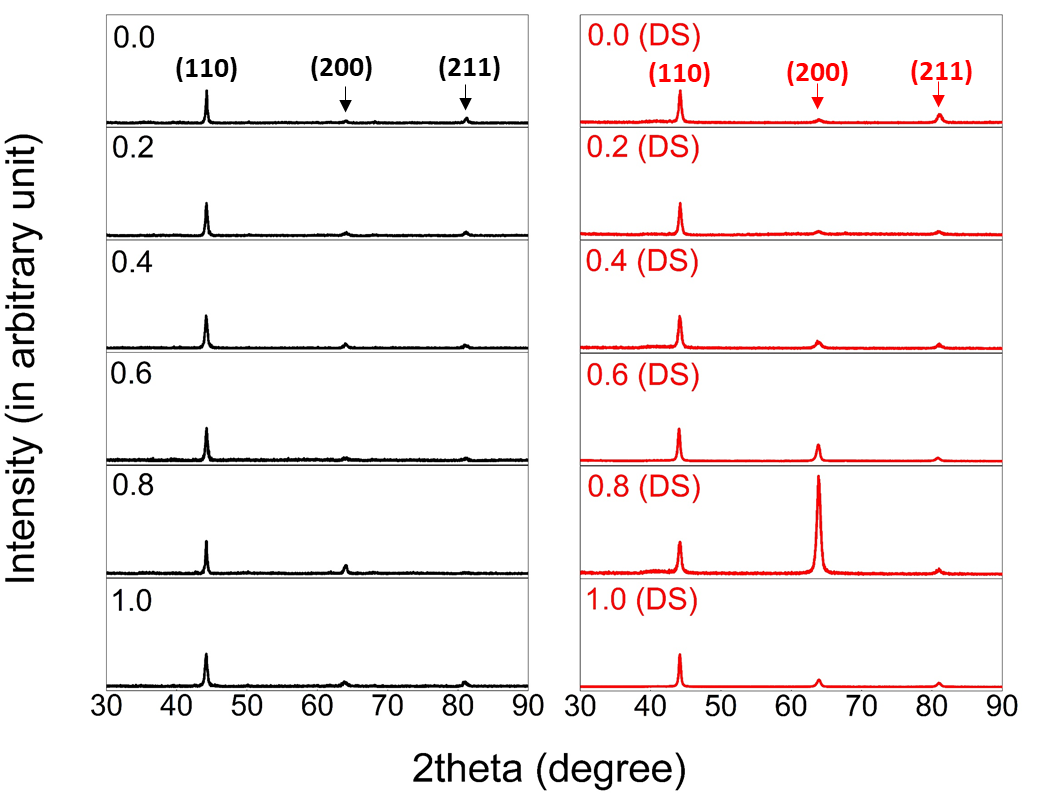
**

**Figure S2**. XRD patterns of as-cast and DS-treated (Fe_0.83_Ga_0.17_)_100-x_Pt_x_ (*x* = 0, 0.2, 0.4, 0.6, 0.8 and 1.0) alloys.

Fig.S2 presents the XRD patterns of the as-cast and DS-treated FeGa-Pt alloys. On one hand, it can be seen that all the samples possess pure A2 structure with typical (110), (200) and (211) peaks, whereas, on the other hand, it can be readily observed that the relative intensity of (200) peak increases from *x* = 0 to *x* = 0.8 for as-cast and DS-treated samples, which suggests the increasing domain orientation preference along [100] direction with the increase of Pt content.

**3. Pt content dependent magnetostriction of FeGa-Pt alloys**

**
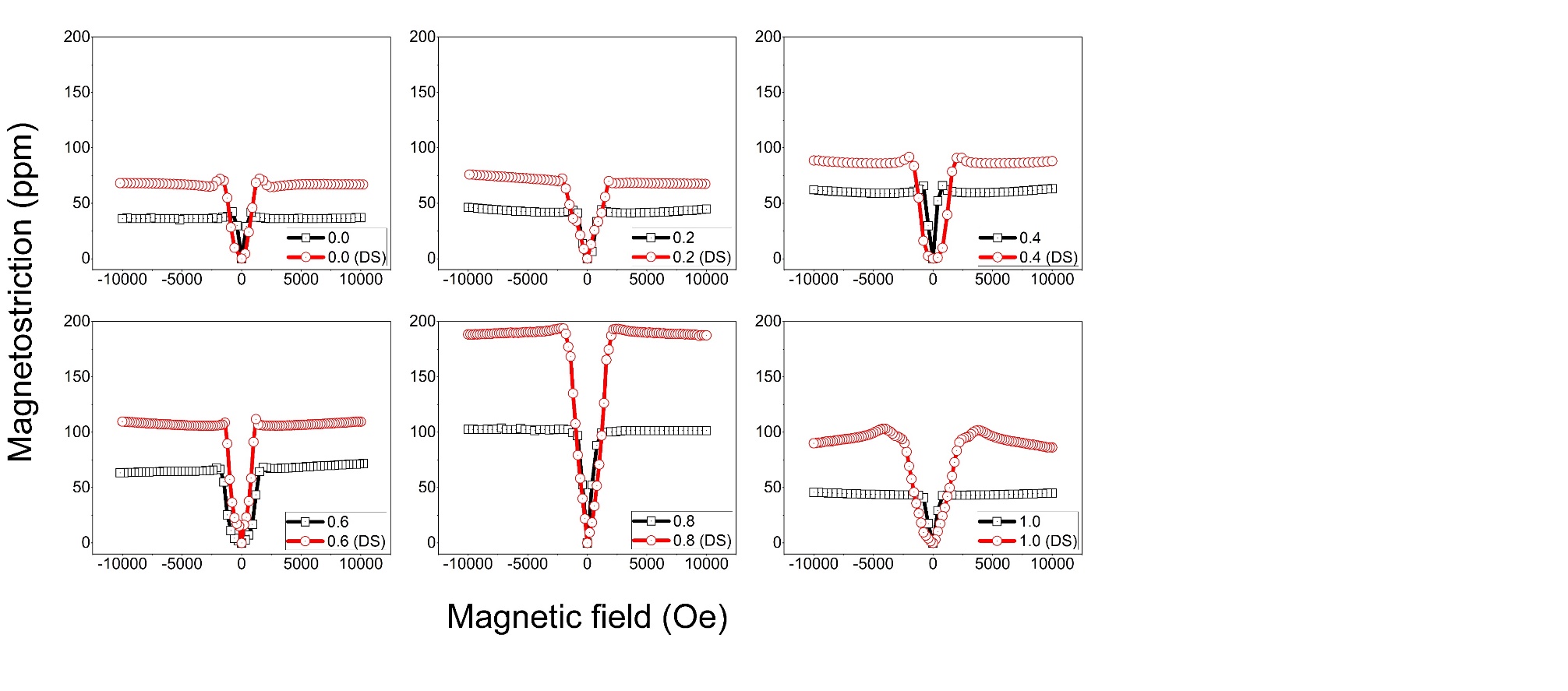
Figure S3**. Magnetostriction curves of as-cast and DS-treated (Fe_0.83_Ga_0.17_)_100-x_Pt_x_ (*x*=0, 0.2, 0.4, 0.6, 0.8 and 1.0) alloys.

Fig.S3 shows the magnetostriction curves of the as-cast and DS-treated FeGa-Pt alloys. In the case of as-cast alloy, with Pt doping, the magnetostriction gradually increases from 39 ppm to 103 ppm for *x* = 0 and 0.8, respectively. In the case of DS-treated samples, the magnetostriction of each composition is obviously improved. It is worth mentioning that the increase of magnetostriction for *x* = 0.8 from 103 ppm to 188 ppm corresponds to an increase of 82.5%, which is the highest among the as-prepared compositions. The composition dependence of magnetostriction is consistent with the intensity ratio between (200) peak and (110) peak and the magneto-crystalline anisotropic constant K_1_.

**4. Calculation of the magneto-crystalline anisotropic constant K_1_**

The calculation of the magneto-crystalline anisotropic coefficient K_1_ was carried out by the method proposed by M. Vazquez et al. (Phys. Stat. Sol. (a) 115, 547 (1989)), which is based on the law of approach to ferromagnetic saturation - a common way to analyze the magnetization curves of soft magnetic polycrystalline materials.

The magneto-crystalline anisotropy constant K_1_ of the Fe-Ga-Pt alloys (Fig. 4c) can be deduced from the following two classical formulas (4) and (5):

 (4)

where *M_S_* refers to the saturation magnetization per unit mass, *a_1_*/*H* represents the contribution from the structural defects (*a_1_* is a constant), *a_2_*/*H*^2^ corresponds to the influence of magneto-crystalline anisotropy (*a_2_* is a constant), and *χ_hf_* denotes the high field susceptibility.

For a ferromagnetic crystal with a cubic structure, the constant *a_2_* can be given as:

$a_{2}=\frac{\frac{8}{105}K_{1}^{2}}{M_{\text{S}}^{2}}$ (5)

where K_1_ denotes the first cubic magneto-crystalline anisotropic constant.

If the terms *a_1_*/*H* and *χ_hf_*·*H* are ignored (J. Alloys compd. 260, 196-200 (1997)), the *M(H)* can be plotted as a function of 1/*H^2^* and the slope after fitting becomes equal to -2*M_S_*·*a_2_*.

For undoped Fe_83_Ga_17_ (*x* = 0, as-cast), from Fig. S4 and the formula (5), *a_2_* is calculated to be 3.08×10^5^ Oe^2^. Then, K_1_ can be obtained from formula (5). The value of K_1_ for as-cast samples increases from 3.72×10^5^ erg/cm^3^ for *x* = 0 to 3.97×10^5^ erg/cm^3^ for *x* = 0.8, and then decreases to 3.89×10^5^ erg/cm^3^ for *x* = 1.0.


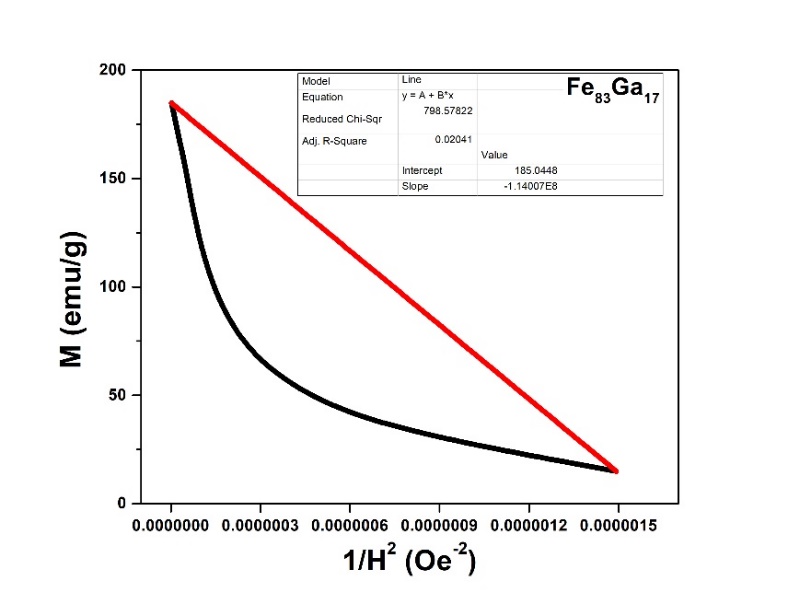


**Figure S4**. The relationship between *M* vs. 1/*H^2^*, fitted with a straight line. The saturation magnetization and the magneto-crystalline anisotropy constant for the as-cast Fe_83_Ga_87_ can be derived from the fitting results.
